# Recombination and pseudorecombination driving the evolution of the begomoviruses *Tomato severe rugose virus* (ToSRV) and *Tomato rugose mosaic virus* (ToRMV): two recombinant DNA-A components sharing the same DNA-B

**DOI:** 10.1186/1743-422X-11-66

**Published:** 2014-04-05

**Authors:** Fábio N Silva, Alison TM Lima, Carolina S Rocha, Gloria P Castillo-Urquiza, Miguel Alves-Júnior, F Murilo Zerbini

**Affiliations:** 1 Departamento de Fitopatologia/BIOAGRO, Universidade Federal de Viçosa, Viçosa, MG 36570-900, Brazil; 2 Current address: FuturaGene Brasil, Avenida José Lembo 1010, Itapeteninga, SP 18210-780, Brazil; 3 Current address: Centro de Investigación Caribia, Corpoica, Santa Marta, Colombia; 4 Current address: Faculdade de Ciências Agrárias, Universidade Federal do Pará, Altamira, PA 68372-040, Brazil

**Keywords:** Geminivirus, Tomato, Genetic variability

## Abstract

**Background:**

Begomoviruses are dicot-infecting, whitefly-transmitted viruses with a genome comprised of one or two molecules of circular, single-stranded DNA. In Brazil, tomato-infecting begomoviruses have emerged as serious pathogens since the introduction of a new biotype of the insect vector in the mid-1990’s. *Tomato rugose mosaic virus* (ToRMV) and *Tomato severe rugose virus* (ToSRV) are often found in tomato fields. The complete sequence of the DNA-B components of ToSRV and ToRMV show an identity of 98.2%. Additionally, the high nucleotide identity (96.2%) between their common regions indicates that these two viruses may share the same DNA-B.

**Methods:**

Tomato seedlings were biolistically inoculated with ToSRV (DNA-A and DNA-B) and ToRMV (DNA-A and DNA-B) infectious clones in every possible combination of single or mixed infection. Symptom expression was evaluated for up to 35 days post-inoculation (dpi). DNA was extracted at 28 dpi and the presence of each viral genomic component was examined by rolling circle amplification (RCA) followed by digestion, as well as by quantitative, real-time PCR. Sequence comparisons, recombination and phylogenetic analyzes were performed using EMBOSS needle, RDP program and maximum likelihood inference, respectively.

**Results:**

Symptoms in tomato plants inoculated with the different combinations of ToRMV and ToSRV DNA-A and DNA-B components consisted of a typical mosaic in all combinations. Pseudorecombinants were formed in all possible combinations. When two DNA-A or two DNA-B components were inoculated simultaneously, the ToRMV components were detected preferentially in relation to the ToSRV components. The combination of minor changes in both the Rep protein and the CR may be involved in the preferential replication of ToRMV components. Recombination and phylogenetic analyzes support the exchange of genetic material between ToRMV and ToSRV.

**Conclusions:**

ToRMV and ToSRV form viable pseudorecombinants in their natural host (*Solanum lycopersicum*) and share the same DNA-B. ToRMV DNA components are preferentially replicated over ToSRV components. These results indicate that the emergence of ToRMV involved both recombination and pseudorecombination, further highlighting the importance of these mechanisms in the emergence and adaptation of begomoviruses.

## Background

The genus *Begomovirus* (family *Geminiviridae*) contains viruses with a genome comprised of circular, single-stranded DNA, transmitted by the whitefly *Bemisia tabaci* (Homoptera: Aleyrodidae) to dicot plant species [1]. Most New World begomoviruses have bipartite genomes consisting of two ssDNA components named DNA-A and DNA-B, each with approximately 2600 nucleotides (nt). These components share a common region (CR) of approximately 200 nt with high sequence identity (>85%, with a few exceptions) [2]. The DNA-A encodes viral proteins associated with replication, control of gene expression, suppression of host defense responses and encapsidation, whereas the DNA-B encodes proteins associated with intra- and intercellular movement [3].

The CR includes the origin of replication as well as conserved sequences that are recognized by the replication-associated protein (Rep) [4,5]. Recognition by Rep is considered to be virus-specific [6], so that Rep only initiates replication of cognate DNA components. However, in some cases the Rep protein encoded by a given DNA-A will recognize the DNA-B of a different virus, a phenomenon known as pseudorecombination [3]. Pseudorecombinants are usually formed with DNA components from strains of the same viral species [7,8], although, more rarely, they can also be formed with components from different species [9-11].

The viability of a pseudorecombinat is, at least partially, a function of the conservation of the high affinity binding sites for the Rep protein. For most begomoviruses these binding sites include two direct repeats and one inverted repeat known as iterons [6]. DNA components with identical iterons will usually form viable pseudorecombinants, whereas those with different iterons will not [12,13]. However, in both cases there are exceptions [9,14], indicating that other, less understood factors also play a role in pseudorecombinant formation. The Rep protein of begomoviruses has specificity determinants (SPDs), which confer specificity to the recognition of iterons [13]. Mutation or deletion of SPDs located adjacent to motif 1, known as the iteron-related domain (IRD) [15], eliminates the ability of the Rep protein to bind DNA in a sequence-specific manner [16,17].

Genetic exchanges through recombination allow for a rapid evolution of plant viruses, in many cases promoting changes in virulence or host range [18]. The role of recombination in the emergence, establishment and evolution of novel, better adapted begomoviruses is well established [19-27]. In Spain, a recombinant between *Tomato yellow leaf curl virus* (TYLCV) and *Tomato yellow leaf curl Sardinia virus* (TYLCSV) displayed a wider host range compared to both parental viruses, and became established in the field [22]. In Uganda, a recombinant between *African cassava mosaic virus* (ACMV) and *East African cassava mosaic virus* (EACMV) displayed increased virulence and aggressiveness, and was responsible for devastating epidemics of cassava mosaic disease during the 1990’s [25,26]. The combined effect of pseudorecombination and recombination in the emergence of novel begomoviruses was elegantly demonstrated by the work of Hou & Gilbertson [28]. Working with *Bean dwarf mosaic virus* (BDMV) and *Tomato mottle virus* (ToMoV), the authors showed that, after five consecutive passages in *Nicotiana benthamiana*, a pseudorecombinant with ToMoV DNA-A and BDMV DNA-B underwent a recombination event in which part of the ToMoV DNA-A CR was transferred to the BDMV DNA-B. This resulted in an marked increase in the titer of the DNA-B and in the induction of more severe symptoms by the pseudorecombinant.

The incidence and severity of begomovirus epidemics in tomatoes has greatly increased in Brazil since the mid-1990’s, following the introduction and dissemination of the B biotype of *B. tabaci*[29]. The initial characterization of begomoviruses associated with these epidemics indicated a high degree of genetic diversity [30,31]. Several new species have been described, including *Tomato rugose mosaic virus* (ToRMV) [32], *Tomato chlorotic mottle virus* (ToCMoV) [33] and *Tomato yellow spot virus* (ToYSV) [34]. Mixed infections, a pre-requisite for the occurrence of recombination and pseudorecombination events, are common in the field [27,35,36]. Our comprehensive analysis of Brazilian begomovirus populations provided strong support for the hypothesis that tomato-infecting begomoviruses evolved from indigenous viral populations present in non-cultivated hosts, and indicated that recombination played a major role in the emergence of these viruses as tomato pathogens [27].

ToRMV and *Tomato severe rugose virus* (ToSRV) are often found in tomato fields in Southeastern Brazil [27,32,35]. The complete DNA-B sequences of ToRMV (GenBank: AF291705) and ToSRV (DQ207749) show an identity of 98.2%, suggesting that these two viruses may share the same DNA-B. Additionally, recombination analysis indicated that part of the CR and most of the Rep gene were transferred from ToSRV to ToRMV [33].

The results presented here indicate that ToRMV and ToSRV isolates are capable of forming viable pseudorecombinants in their natural host, tomato (*Solanum lycopersicum*). Symptom severity was equivalent in single or mixed infections, indicating that synergism does not occur between these two viruses. However, ToRMV DNA-A and -B components were preferentially replicated over ToSRV components. These results further highlight the importance of recombination and pseudorecombination in the emergence and adaptation of begomoviruses, and pose a challenging taxonomical question: To what extent should a recombinant be considered a new species?

## Results

### Latent period, symptoms and infectivity of the different combinations between ToRMV and ToSRV DNA-A and DNA-B

**Figure 1:**
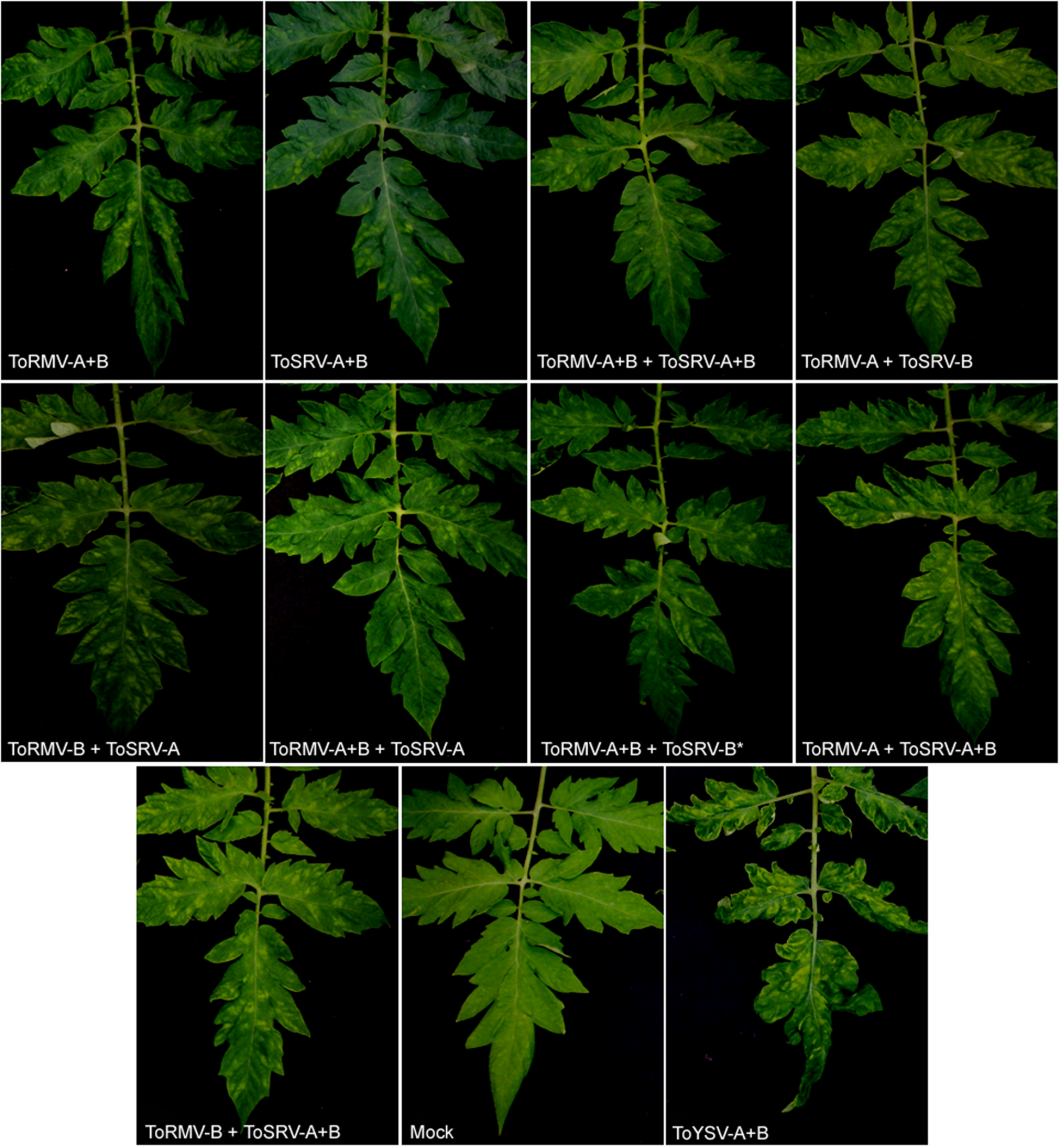
**Symptoms induced in tomato (*Solanum lycopersicum *‘Santa Clara’) biolistically inoculated with the infectious clones of the DNA-A and -B of isolates ToRMV-[BR:Ub1:96] and ToSRV-[BR:PG1:Pep:03].** The different combinations of ToRMV and ToSRV DNA-A and DNA-B components which were inoculated are indicated. All images obtained at 28 dpi. *ToSRV-B was not detected in assessed plants.

Symptoms in tomato plants inoculated with the different combinations of ToRMV and ToSRV DNA-A and DNA-B first appeared at 14 dpi (Table 1), and consisted of a typical mosaic in all combinations (Figure 1). In plants inoculated with ToYSV (used as a positive control) the first symptoms appeared at 10 dpi (Table 1), and included yellow mosaic, yellow spots and leaf distortion (Figure 1). The presence of each inoculated DNA component was verified by RCA followed by digestion with component-specific enzymes (Table 1 and Additional file 1: Figure S1). In single infections, infectivity of the two homologous combinations (ToRMV and ToSRV) was 92% and 100%, respectively. Pseudorecombinants were formed in both possible combinations with high infectivity (100% and 92%). Interestingly, when two DNA-A or two DNA-B components were inoculated simultaneously, the ToRMV component was detected preferentially in relation to the ToSRV component (Table 1, underlined values; Additional file 1: Figure S1). This was true even in the two cases in which ToSRV DNA-A and -B were inoculated with either ToRMV DNA-A or -B, suggesting that the Rep protein encoded by ToSRV may have a higher affinity for the ToRMV binding sites. Moreover, when all four components were inoculated, the two ToRMV components were detected in 100% of the plants, but the ToSRV components were detected in only 15% and 8% of the plants (DNA-A and DNA-B, respectively), again suggesting a preferential replication of ToRMV DNA components over ToSRV components.

**Table 1:** Infectivity and latent period of pseudorecombinants formed between ToRMV-[BR:Ub1:96] and ToSRV-[BR:PG1:Pep:03] in tomato plants

**Combination**	**Latent period (days)**^**a**^	**Infected/Inoculated (%)**^**b**^				
		**ToRMV-A**	**ToRMV-B**	**ToSRV-A**	**ToSRV-B**	**ToYSV**
ToRMV-A + B	14	11/12(92)	11/12(92)	-	-	-
ToSRV-A + B	14	-	-	13/13(100)	13/13(100)	-
ToRMV-A + ToSRV-B	14	13/13(100)	-	-	13/13(100)	-
ToRMV-B + ToSRV-A	14	-	12/13(92)	12/13(92)	-	-
ToRMV-A + B + ToSRV-A	14	12/13(92)	12/13(92)	*1/13(8)*^c^	-	-
ToRMV-A + B + ToSRV-B	14	10/13(77)	10/13(77)	-	*0/13(0)*	-
ToRMV-A + ToSRV-A + B	14	11/12(92)	-	*1/12(8)*	12/12(100)	-
ToRMV-B + ToSRV-A + B	14	-	12/13(92)	12/13(92)	*4/13(31)*	-
ToRMV-A + B + ToSRV-A + B	14	13/13(100)	13/13(100)	*2/13(15)*	*1/13(8)*	-
ToYSV-A + B	10	-	-	-	-	9/9(100)

^a^Number of days elapsed between inoculation and the appearance of symptoms.

^b^Number of infected plants/number of inoculated plants, verified by visual observation of symptoms and confirmed by RCA following digestion with component-specific restriction enzymes. The total number of inoculated plants represents two independent experiments.

^c^Values in italics indicate the low detection of ToSRV components when inoculated simultaneously with ToRMV components.

**Figure 2:**
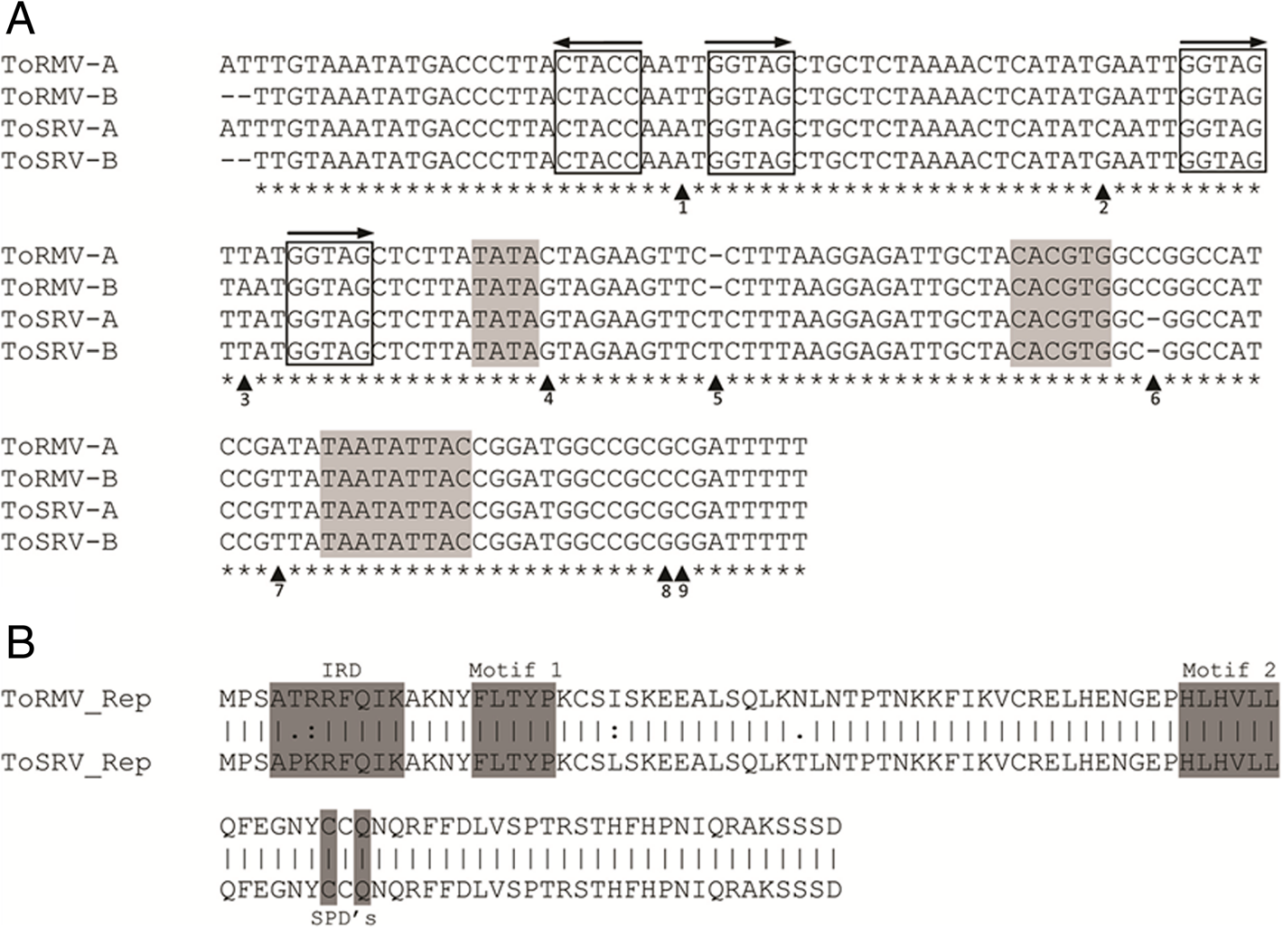
**Alignment of the common regions and the Rep proteins of ToSRV-[BR:PG1:Pep:03] and ToRMV-[BR:Ub1:96]. A**. Common region alignment. The TATA box, G-box and the conserved nonanucleotide are indicated in light gray. Iterated direct and inverted repeats (iterons) are boxed. The arrows indicate the direction of the repeats. Asterisks indicate nucleotide positions which are conserved among all four aligned sequences. Arrowheads indicate differences in nucleotide sequence among the four CRs. **B**. Rep protein alignment. Dark gray boxes correspond to the domain associated with sequence-specific recognition of iterons (iteron-related domain, IRD), motif 1, motif 2 and specificity determinants (SPDs), which according to [15] and [37] are conserved in rolling-circle replication-initiator proteins, including geminivirus Rep proteins.

These results are striking, considering the extremely high identity between the DNA-B components of ToRMV and ToSRV, including the CR (which have identical iterons) (Table 2 and Figure 2A).

**Table 2:** Percent nucleotide and deduced amino acid sequence identities between the full length DNA components and each open reading frame of ToRMV-[BR:Ub1:96] and ToSRV-[BR:PG1:Pep:03]

**Identity**	**DNA**			**Open reading frames**^**2**^						
	**A**	**B**	**CR**^**1**^	**Rep**	**TrAP**	**REn**	**AC4**	**CP**	**MP**	**NSP**
Nucleotide	86.3	98.2	95	95.3	83.1	86.6	97.0	81.1	99.1	98.7
Amino acid	-	-	-	93.5	77.7	81.2	90.8	90.1	99.0	97.7

^1^CR, common region.

^2^Rep, replication-associated protein; TrAP, transcriptional activator protein; REn, replication enhancer protein; CP, coat protein; MP, movement protein; NSP, nuclear shuttle protein.

To confirm the negative interference of ToRMV over ToSRV, primers for quantitative, real-time PCR (qPCR) were designed based on the most divergent region of the DNA-A of each virus. The primers designed for the detection of ToRMV DNA-A were not specific, yielding multiple, non-specific fragments from ToRMV-infected samples (data not shown). Therefore, only ToSRV DNA-A accumulation was quantified in single or dual infected plants (in which both DNA-A’s were co-inoculated). The qPCR assay revealed a strong reduction of ToSRV DNA-A accumulation in plants co-inoculated with ToRMV DNA-A (Additional file 2: Figure S2). Due to the high nucleotide sequence identity between the DNA-B’s of both viruses (98.2%), it was not possible to design DNA-B-specific primers. However, the RCA-RFLP assay for the DNA-B strongly suggests a similar pattern to that of DNA-A accumulation.

Together, these results indicate that ToSRV and ToRMV form viable pseudorecombinats in tomato plants and that synergism does not occur between these two viruses. Quite the contrary, probably ToRMV has a negative interference over ToSRV, although ToSRV can replicate and induce symptoms as efficiently as ToRMV in a single infection.

### Nucleotide and deduced amino acid sequence comparisons

In an attempt to identify genomic differences that may explain the negative interference of ToRMV over ToSRV, comparisons of their coding and non-coding regions were carried out. The CRs of the two viruses show sequence identities ranging from 95% (ToSRV-A vs. ToRMV-B, and ToSRV-B vs. ToRMV-A) to 97.5% (ToSRV-A vs. -B), with identical iterons (Figure 2A). Alignment of the four CRs indicated nine positions with nucleotide sequence divergence (Figure 2A). At divergent positions 1, 5 and 6, the sequence is conserved between the cognate DNA components of ToRMV and ToSRV. At other positions one component differs from the other three: ToRMV-A at positions 4 and 7, ToRMV-B at positions 3 and 8, ToSRV-A at position 2, and ToSRV-B at position 9 (Figure 2A).

The comparison of nucleotide and amino acid sequences of the Rep gene indicates identities of 95.3% and 93.5%, respectively (Table 2). This was expected since the region involved in the recombination event comprises practically the entire Rep gene. The domains associated with sequence-specific recognition of the origin of replication, named “iteron-related domain” (IRD) [15], “Motif 1”, “Motif 2” and specificity determinants (SPDs) [37], conserved in rolling-circle replication-initiator proteins including geminivirus Rep proteins, are shown in Figure 2B. ToRMV has four amino acid changes compared with ToSRV. Two changes are in the IRD (Pro → Thr and Lys → Arg). These differences are also present in 85 aditional isolates which have been sequence in our laboratory over the years (data not shown).

### Recombination analysis

To further investigate the occurrence of recombination events involving ToSRV and ToRMV, recombination analysis was carried out using the RDP3 package. Besides ToRMV and ToSRV, the analysis included seventeen closely related begomoviruses that occur in cultivated and non-cultivated plants (Table 3).

**Table 3:** Begomoviruses used in the recombination and phylogenetic analyses involving ToRMV and ToSRV

**Virus**	**GenBank access number (DNA-A)**
*Abutilon Brazil virus* (AbBV)	FN434438
*Bean golden mosaic virus* (BGMV)	M88686
*Blainvillea yellow spot virus* (BlYSV)	EU710756
*Cleome leaf crumple virus* (ClLCrV)	FN435999
*Okra mottle virus* (OMoV)	EU914817
*Sida common mosaic virus* (SiCmMV)	EU710751
*Sida micrantha mosaic virus* (SimMV)	AJ557451
*Sida mottle virus* (SiMoV)	AY090555
*Sida yellow leaf curl virus* (SiYLCV)	EU710750
*Sida yellow mosaic virus* (SiYMV)	AY090558
*Tomato golden mosaic virus* (TGMV)	K02029
*Tomato chlorotic mottle virus* (ToCMoV)	AF490004
*Tomato common mosaic virus* (ToCmMV)	EU710754
*Tomato leaf distortion virus* (ToLDV)	EU710749
*Tomato mild mosaic virus* (ToMlMV)	EU710752
*Tomato rugose mosaic virus* (ToRMV)	AF291705
*Tomato severe rugose virus* (ToSRV)	DQ207749
*Tomato yellow spot virus* (ToYSV)	DQ336350
*Tomato yellow vein streak virus* (ToYVSV)	EF417915

**Figure 3:**
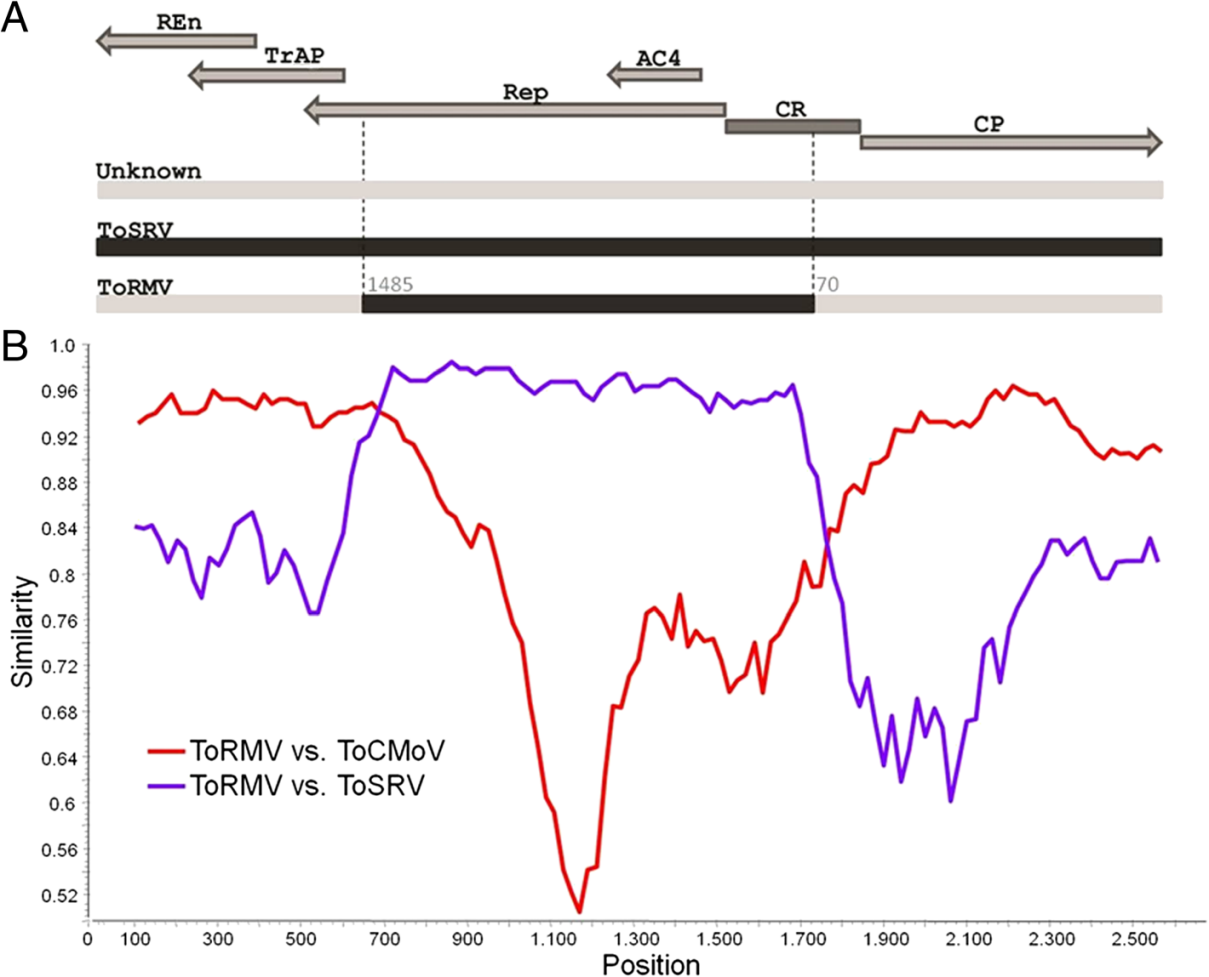
**A putative recombination event involving ToRMV-[BR:Ub1:96] and ToSRV-[BR:PG1:Pep:03]. A**. Schematic representation of the recombinant region between ToSRV and ToRMV. Arrows indicate coding regions in the complementary (AC4, Rep, Trap and Ren) and viral sense (CP) genes. CR indicates the common region. The dashed line indicates the position of putative breakpoints of the recombinant region. **B**. Similarity plot for ToRMV against ToSRV and ToCMoV along a 200-nt sliding window. The scale in the X-axys corresponds to the viral genome as depicted in **(A)**.

A recombination event involving ToSRV and ToRMV previously proposed by Ribeiro et al. [33] was confirmed by six detection methods contained in the RDP3 package (*P*-values: rdp = 1.667 × 10^−14^, Geneconv = 1.405 × 10^−25^, Bootscan = 3.538 × 10^−28^, maximum χ^2^ = 1.024 × 10^−20^, Chimaera = 2.975.45 × 10^−12^ and Sister scan = 1.171 × 10^−38^). The transferred region comprises practically the entire Rep gene as well as part of the CR including the iterons (Figure 3). Also as previously reported [38], a recombinant event was detected in which ToCMoV received a fragment of ToRMV DNA-A comprising the entire CP and Ren genes, as well as most of the Trap gene. This event was detected by all detection methods (*P*-values: rdp = 3.039 × 10^−39^, Geneconv = 3.734 × 10^−37^, Bootscan = 1.157 × 10^−40^, maximum χ^2^ = 2.085 × 10^−21^, Chimaera = 3.101 × 10^−25^, Sister scan = 4.771 × 10^−43^ and 3Seq = 5.053 × 10^−64^).

### Phylogenetic analysis

**Figure 4:**
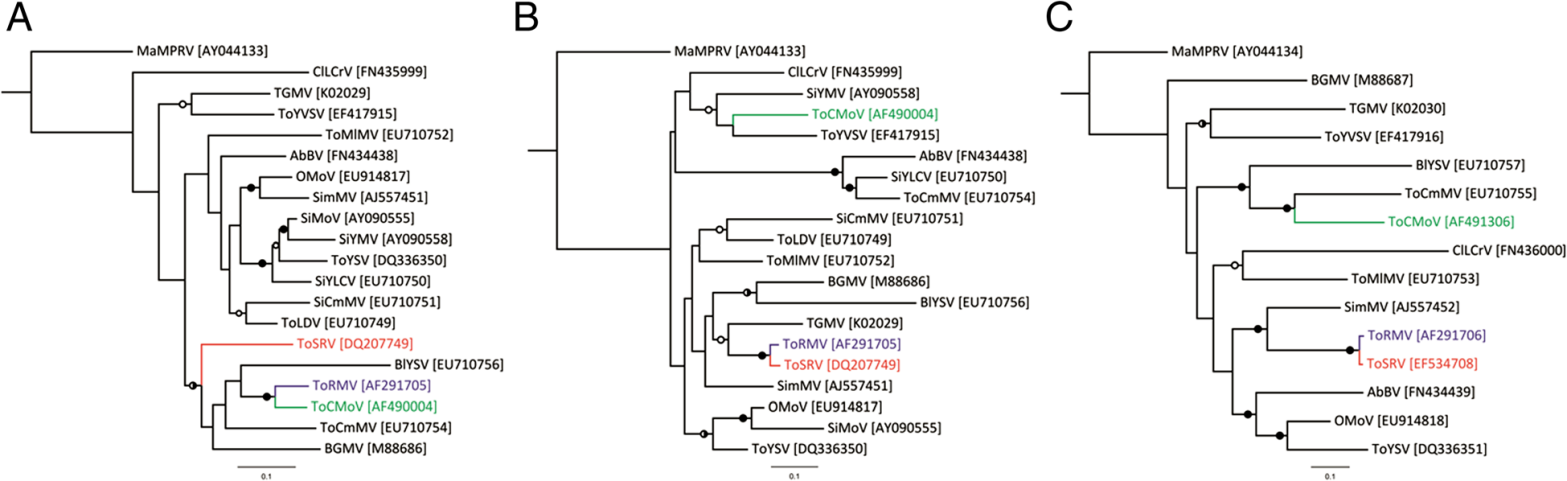
**Maximum likelihood (ML) phylogenetic relationships of ToRMV-[BR:Ub1:96], ToSRV-[BR:PG1:Pep:03] and some closely related begomoviruses. A**. ML tree based on the non-recombinant region between ToRMV and ToSRV in the DNA-A. **B**. ML tree based on the recombinant region between the two viruses in the DNA-A. **C**. ML tree based on full-length DNA-B sequences. Phylogenetic reconstruction was performed using PAUP* 4.0 [58] under the evolutionary models TVM + I + Γ (non-recombinant block), GTR + I + Γ (recombinant block), TIM + I + Γ (DNA-B). Supports for the nodes are presented as filled circles (bootstrap values from 95 to 100), semi-filled circles (bootstrap values from 85 to 94) and open circles (boostrap values from 70 to 84). ToRMV and ToSRV are indicated in blue and red, respectively. ToCMoV is indicated in green.

Phylogenetic analysis based on non-recombinant and recombinant nucleotide sequences between ToRMV and ToSRV in the DNA-A demonstrates topological incongruence, supporting the exchange of genetic material between the two viruses. ToRMV was placed in the same branch with ToCMoV in the tree based on the non-recombinant region (Figure 4A) and with ToSRV in the tree based on the recombinant region (Figure 4B), in both cases with high bootstrap values, indicating a close phylogenetic relationship. In the phylogenetic tree based on the DNA-B, ToRMV and ToSRV formed a well-supported clade with branches of the same size and a bootstrap value of 100% (Figure 4C). In addition, the high identity between their DNA-B sequences (nt and deduced aa; Table 2) suggests that the capture of the ToSRV DNA-B by ToRMV was a recent event.

## Discussion

Here, we report the formation of pseudorecombinants between two distinct, albeit closely related, begomoviruses. The reciprocal pseudorecombinants ToRMV-A + ToSRV-B and ToRMV-B + ToSRV-A were both viable. Their viability is most likely due to the high sequence identity of their CRs and Rep proteins, as well as the identical sequences of their iterons (GGTAG). The latent period and symptoms were the same in all combinations compared to the parental viruses, which is not surprising since the severity of symptoms is associated with the DNA-B, and these are almost identical between the two viruses.

When DNA-A and DNA-B components of both ToSRV and ToRMV were inoculated, mosaic symptoms identical to those induced by each virus alone appeared at 14 dpi (Figure 1 and Table 1). This suggests that synergism does not occur between these two viruses. On the contrary, ToRMV had a negative interference over ToSRV. In most cases in which three or four DNA components were inoculated, the ToRMV components were always detected, whereas the ToSRV components were rarely detected. Negative interference exerted by ToRMV in mixed infection with ToYSV has already been reported [39]. Early in infection ToRMV negatively affected the accumulation of ToYSV, but when the infection was established the negative interference ceased [39]. Interestingly, although ToRMV negatively affected ToSRV in our experiments, ToSRV behaved similarly to ToRMV in a single infection, both in terms of latent period and symptoms.

Considering that their DNA-B components are almost identical, and that their iterons are identical, we hypothesized that the preferential replication of ToRMV over ToSRV would be due to differences in their Rep proteins, most likely in their virus-specific recognition domains. Begomovirus iterons are composed of an invariable GG sequence followed by three nt (identified as N1, N2 and N3) that vary for each virus. The virus-specific recognition domain of the Rep protein maps to its N-terminal region [40,41] and includes the conserved motif 1 of rolling-circle replication-initiator proteins [42]. The IRD sequence is conserved among begomoviruses with identical iterons, but varies among species with different iteron sequences. Predicted nt-aa pairing would occur between N1 and the eighth aa of the IRD, N2 with the sixth IRD aa, and N3 with the first or third IRD aa, depending on the iteron sequence [15].

Although ToRMV and ToSRV have identical iterons, their IRDs are different at positions 2 and 3. The change of lysine to arginine at IRD position 3, which pairs with N3, should not promote major changes in the protein, since the two amino acids have similar properties. Therefore, it is reasonable to assume that this difference is not responsible for the preferential replication of ToRMV over ToSRV. In fact, this change does not affect iteron recognition, since the two Rep proteins have the ability to replicate viral DNA. This is observed when any combination of a DNA-A and a DNA-B component (cognate or pseudorecombinant) is inoculated: both components are always detected (Table 1).

Further analysis revealed small differences between ToRMV and ToSRV in the CR and in the Rep protein. In the CR, nine nucleotide changes occur among the four components, with ToRMV DNA components having the largest number of modifications compared to ToSRV. It is possible that these changes in the CR sequence, as well as changes in IRD aa 2 and 3, have enabled the ToRMV Rep protein to recognize the iteron sequence GGTAG with higher affinity compared to ToSRV. These same differences could cause the ToSRV Rep protein to replicate the DNA-A and -B of ToRMV preferentially. More likely is that the combination of minor changes in both Rep and CR may be involved in the preferential replication of ToRMV components. A detailed mutagenesis analysis of the IRD region and of the iterons of ToRMV and ToSRV will be required to confirm this hypothesis.

Interestingly, ToSRV is more prevalent in the field compared to ToRMV [27,35]. This could be due to differences in transmission by the insect vector, since the coat protein, which is considerably distinct between the two viruses, is the major viral determinant of insect transmission.

The detection of a recombination event involving the DNA-A of the two viruses was not surprising, since it is well established that begomoviruses are recombination-prone. The region transferred from ToSRV to ToRMV comprises practically the entire Rep ORF as well as part of the CR including the TATA box, G-box, the conserved nonanucleotide (TAATATTAC) and the iterons. This recombination event between ToSRV and ToRMV was detected also by Ribeiro et al. [33], who proposed that ToRMV would be a recombinant between ToCMoV and ToSRV.

The specific genomic region involved in a recombination event is an important aspect in the maintenance and adaptation of the recombinant virus in the population. It is known that the begomovirus genome has a number of recombination hot spots, including the CR, the CP/Ren interface and the 3′ region of the Rep gene, while the remaining coding regions are recombination cold spots [28,43-45]. The breakpoints of the recombination event involving ToSRV and ToRMV map to two hotspots, the CR and to the 3′ region of the Rep gene, near the interface with the Trap gene. Recombination in coding regions probably occurs due to the conflict between the machineries of transcription and replication [45,46]. Lefeuvre et al. [47] suggest that selection apparently disfavors recombinants with breakpoints in coding regions, and that the probability of such a recombinant to remain in the population is dependent on the specific position of the breakpoint: the peripheries of coding regions tend to be more favorable to recombinantion than central regions. However, recent experimental evidence demonstrated that the coding regions of the begomoviruses *Tomato yellow leaf curl virus*-Mld (TYLCV) and *Tomato leaf curl Mayotte virus* (ToLCYTV) are recombination prone, and that all recombinants between TYLCV and ToLCYTV generated using L-DNA-shuffling technology displayed infectivity and viral accumulation similar to the parental begomoviruses [48].

Recombination events involving DNA-A components are considered a major source of molecular variation for begomoviruses and may result in a gain of virulence [49,50]. However, predicting the epidemiological outcome of a recombination event is not a straightforward task. For example, van der Walt et al. [24] demonstrated that infection by a recombinant between different MSV isolates led to increased symptom severity in maize, indicating greater adaptation of the recombinant. On the other hand, Davino et al. [21] demonstrated that a recombinant between TYLCV and TYLCSV coexists with the parental viruses and has a lower replicative capacity, a fact that might help to explain why plants infected only by the recombinant have not been found. In the case of the recombinant ToRMV, there was an adaptive advantage over the parental ToSRV reflected in the preferential replication of ToRMV over ToSRV, nevertheless ToSRV is more prevalent in the field compared to ToRMV. We suggest that ToSRV is maintained in nature by infecting plants in the absence of ToRMV. If the occurrence of mixed infection between ToRMV and ToSRV was frequent, ToRMV would be the prevalent virus.

Another interesting aspect is that the two DNA-B components are almost identical (98.2% nt identity). This supports the hypothesis that, following the transfer of practically the entire Rep ORF as well as part of the CR from ToSRV to ToRMV, ToRMV captured the DNA-B of ToSRV. According to the current ICTV-supported criteria for species demarcation, a begomovirus is considered to represent a new species when its DNA-A sequence is less than 89% identical to that of other begomoviruses [51]. ToSRV and ToRMV DNA-A components have nucleotide sequence identity of 86.3%, which clearly separates them as members of different species based on this criterion. However, these two viruses are very similar in terms of biological and molecular properties, including: (*i*) high sequence identity of their CRs and their Rep coding regions; (*ii*) nearly identical Rep binding sites in their CRs, with identical iterons; (*iii*) the same DNA-B; and (*iv*) induction of nearly identical symptoms in tomato, with the same latent period. We thus propose that ToRMV and ToSRV should be considered to be strains of the same viral species, named ToRMV since this virus was described first.

The fact that begomovirus taxonomy ignores the DNA-B was addressed by Briddon et al. [2], who performed a hypothetical taxonomy based on the DNA-B sequence alone. The authors show that 85% of the sequences analyzed remain unaltered in their classification based on the DNA-A, providing good support for a taxonomy based only on the DNA-A. However, in specific cases such as the one presented in this work, the criterion based on the DNA-A could be less restrictive and/or the DNA-B could be considered in the analysis for species assignment.

## Conclusions

The recombination analysis together with the formation of viable pseudorecombinants between ToSRV and ToRMV support the hypothesis that ToRMV captured the DNA-B of ToSRV after acquiring, by recombination, the DNA-A region that includes the Rep ORF and the CR. Therefore, the emergence of ToRMV involves both recombination and pseudorecombination events with ToSRV. We propose that ToRMV and ToSRV should be considered to be members of the same viral species, retaining the name ToRMV since it was first described.

## Methods

### Pseudorecombination between ToRMV and ToSRV

Full-length DNA-A and DNA-B clones of the viral isolates ToRMV-[BR:Ub1:96] [32] and ToSRV-[BR:PG1:Pep:03] [52] were used in the experiments. Tomato (*S. lycopersicum* cv. ‘Santa Clara’) seedlings were biolistically inoculated [53] using two micrograms of each genomic component (DNA-A and DNA-B) in every possible combination of single or mixed infection. Inoculated plants were kept in a greenhouse with average daily temperatures of 26 ± 2°C. Two independent experiments were carried out, with six plants inoculated for each treatment in the first experiment, and seven in the second experiment.

Inoculated plants were evaluated for symptom expression for up to 35 days post-inoculation (dpi). DNA from all plants was extracted at 28 dpi as described [54] and the presence of each viral genomic component was examined by rolling circle amplification (RCA) [55], followed by digestion with component specific restriction enzymes: *Xho*I and *Bgl*II to detect ToRMV-A and -B, respectively; *Hin*dIII and *Bgl*II + *Sac*II to detect ToSRV-A and -B, respectively.

### Quantification of ToRMV and ToSRV by quantitative, real time PCR (qPCR)

Based on the results obtained from RCA-RFLP assays, control plants (mock-inoculated, inoculated with ToRMV DNA-A + DNA-B and inoculated with ToSRV DNA-A + DNA-B) and plants in which the DNA-A’s of both viruses were co-inoculated in different combinations (ToRMV DNA-A + DNA-B and ToSRV DNA-A + DNA-B; ToRMV DNA-A + DNA-B and ToSRV DNA-A; ToRMV DNA-A and ToSRV DNA-A + DNA-B) were selected for quantitative, real-time PCR (qPCR) assays. Primers were designed based on the sequence of the common region of the DNA-A (in the most divergent region between the two viruses) using the qPCR tool implemented in http://www.idtdna.com/scitools/Applications/RealTimePCR: ToRMV-A(For), 5′CAT CGG GCC TCT GTT GG3′ and ToRMV-A (Rev), 5′GTT ATG CAA CTT GGG CGT TAA G3′; ToSRV-A(For), 5′AAA GTA AAG TGA TTG TCT GTG G3′ and ToSRV-A(Rev), 5′GCC GTT CAA CAA ATT GGG3′. Primer specificity was tested by conventional PCR using positive samples for each virus, followed by electrophoresis in 2.0% agarose gel. Reactions were prepared in a final volume of 10 μL, using Fast SYBR Green Master Mix (Applied Biosystems) following the manufacturer’s instructions, and were analyzed in a CFX96 Real-Time PCR System (Bio-Rad). The PCR protocol included an initial denaturing step at 95°C for 20s, followed by 39 cycles of 95°C for 3 s and 60°C for 30s, followed by a dissociation stage.

Standard curves were prepared using serial dilutions of plasmid DNA containing an insert corresponding to the complete ToSRV DNA-A (10° to 10^6^ copies of the viral genome per reaction). Standard curves were obtained by regression analysis of cycle threshold (Ct) values of each one of the three replications of a given dilution in relation to the log of the amount of DNA in each dilution. For absolute quantification of the number of viral DNA molecules in the different treatments, 100 ng of total DNA was extracted as described previously and used in reactions containing virus-specific primers. Each sample was analyzed in triplicates, and three different plants of each treatment described above were assayed. DNA-B accumulation was not evaluated due to the high sequence similarity between the two viruses, which prevented the design of virus-specific primers for the qPCR assay.

### Sequence comparisons and recombination analysis

Complete genomes, individual coding regions and the CRs of ToRMV and ToSRV were compared using EMBOSS pairwise alignment algorithms (http://www.ebi.ac.uk/Tools/emboss/align/index.html), using the EMBOSS needle (Global) with default settings. Nineteen New World begomovirus DNA components (Table 3) that occur in weeds and tomato plants in Brazil were aligned for analysis of recombination events involving ToRMV and ToSRV, using RDP3 [56]. Recombination events detected by at least four of the analysis methods available in the program were considered reliable. Alignments were scanned using default settings for each analysis method using a Bonferroni-corrected *P* value cutoff of 0.05.

### Phylogenetic analysis

Multiple sequence alignments were performed for the DNA-A sequences of Brazilian begomoviruses (Table 3) based on the recombinant and non-recombinant regions of the ToRMV genome (determined in RDP3) and for the cognate full-length DNA-B sequences using Muscle [57]. Maximum likelihood (ML) trees were inferred using PAUP v. 4.0 [58] with the best fit model of nucleotide substitution determined using Modeltest [59] by the Akaike Information Criterion (AIC). A heuristic search was initiated with a neighbor-joining tree using the tree-bisection-reconnection (TBR) algorithm to optimize the ML tree. The robustness of each internal branch was estimated from 3,000 bootstrap replications. The Nearest Neighbor Interchange (NNI) algorithm was used to optimize the bootstrap replications. Trees were visualized and edited using the FigTree (http://www.tree.bio.ed.ac.uk/software/figtree). The begomovirus *Macroptilium mosaic Puerto Rico virus* (MaMPRV) [GenBank: AY044133] was used as an outgroup.

## Competing interests

The authors declare that they have no competing interests.

## Authors’ contributions

FMZ, FNS and ATML designed the study. FNS, ATML, GPCU and MAJ performed the experiments. FNS, ATML and CSR analyzed the data. FNS and FMZ wrote the manuscript. All authors have read and approved the final version of the manuscript.

## Supplementary Material

Additional file 1: Figure S1 Detection by rolling circle amplification followed by restriction digests (RCA-RFLP) of each DNA component from ToRMV-[BR:Ub1:96] and ToSRV-[BR:PG1:Pep:03] after biolistic inoculation of tomato plants. **A**. Digestion with *Hin*d III, generating a fragment of approximately 2600 nucleotides (nt) corresponding to ToSRV DNA-A (the other three components are not cleaved by this enzyme). **B**. Digestion with *Xho* I generating a fragment of approximately 2600 nt corresponding to ToRMV DNA-A (the other three components are not cleaved by this enzyme). **C**. Digestion with *Bgl* II + *Sac* II, generating fragments of 1800 and 800 nt corresponding to ToSRV DNA-B, and approximately 2600 nt corresponding to ToRMV DNA-B (the two DNA-A components are not cleaved by these enzymes). Underlined means DNA-A + DNA-B. (–) negative control. M, 1 kb plus DNA ladder (Invitrogen), in bp. Click here for file

Additional file 2: Figure S2 ToSRV-[BR:PG1:Pep:03] DNA-A accumulation in single or dual infections with ToRMV-[BR:Ub1:96] in tomato plants. Total DNA was extracted from systemically infected leaves at 28 days post-inoculation and used as a template for quantitative, real-time PCR (qPCR) using ToSRV DNA-A primers. 1. Mock-inoculated plants; 2. ToRMV DNA-A + DNA-B; 3. ToSRV DNA-A + DNA-B; 4. ToRMV DNA-A + DNA-B and ToSRV DNA-A + DNA-B; 5. ToRMV DNA-A + DNA-B and ToSRV DNA-A; 6. ToRMV DNA-A and ToSRV DNA-A + DNA-B. In treatment 2, the ToSRV primers weakly amplified ToRMV DNA-A. Values are given as the mean ± confidence interval of three independent tomato plants. Asterisks indicate statistically significant differences by the t test at *P* ≤ 0.05. Click here for file
