# Supplementary figures and images for: Recombination and pseudorecombination driving the evolution of the begomoviruses Tomato severe rugose virus (ToSRV) and Tomato rugose mosaic virus (ToRMV): two recombinant DNA-A components sharing the same DNA-B
Source: Virol J. 2014 Apr 5;11:66. doi: 10.1186/1743-422X-11-66 (PMC4113279; doi:10.1186/1743-422X-11-66)

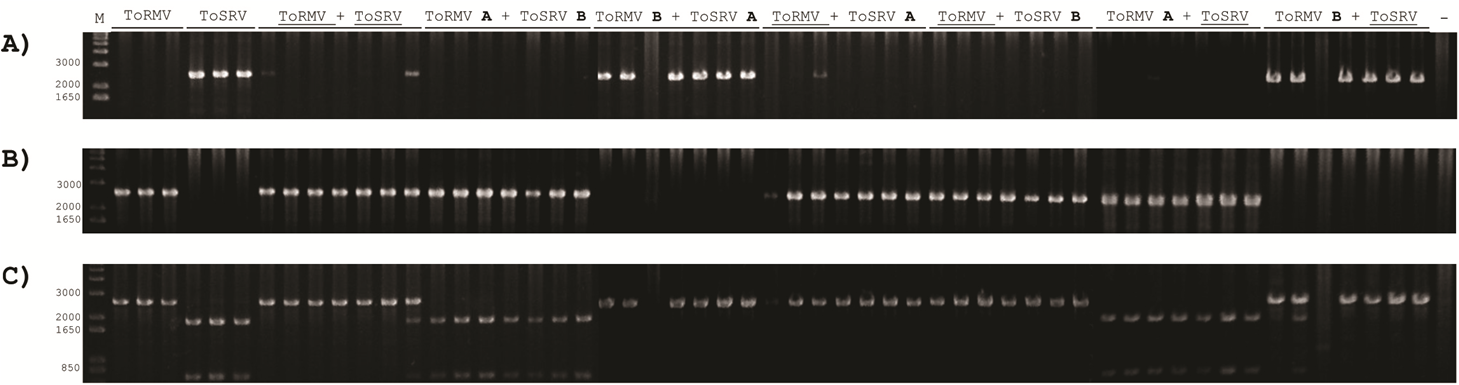

Supplement: Additional file 1: Figure S1 — Detection by rolling circle amplification followed by restriction digests (RCA-RFLP) of each DNA component from ToRMV-[BR:Ub1:96] and ToSRV-[BR:PG1:Pep:03] after biolistic inoculation of tomato plants. A. Digestion with Hind III, generating a fragment of approximately 2600 nucleotides (nt) corresponding to ToSRV DNA-A (the other three components are not cleaved by this enzyme). B. Digestion with Xho I generating a fragment of approximately 2600 nt corresponding to ToRMV DNA-A (the other three components are not cleaved by this enzyme). C. Digestion with Bgl II + Sac II, generating fragments of 1800 and 800 nt corresponding to ToSRV DNA-B, and approximately 2600 nt corresponding to ToRMV DNA-B (the two DNA-A components are not cleaved by these enzymes). Underlined means DNA-A + DNA-B. (–) negative control. M, 1 kb plus DNA ladder (Invitrogen), in bp. [file 1743-422X-11-66-S1.tiff]

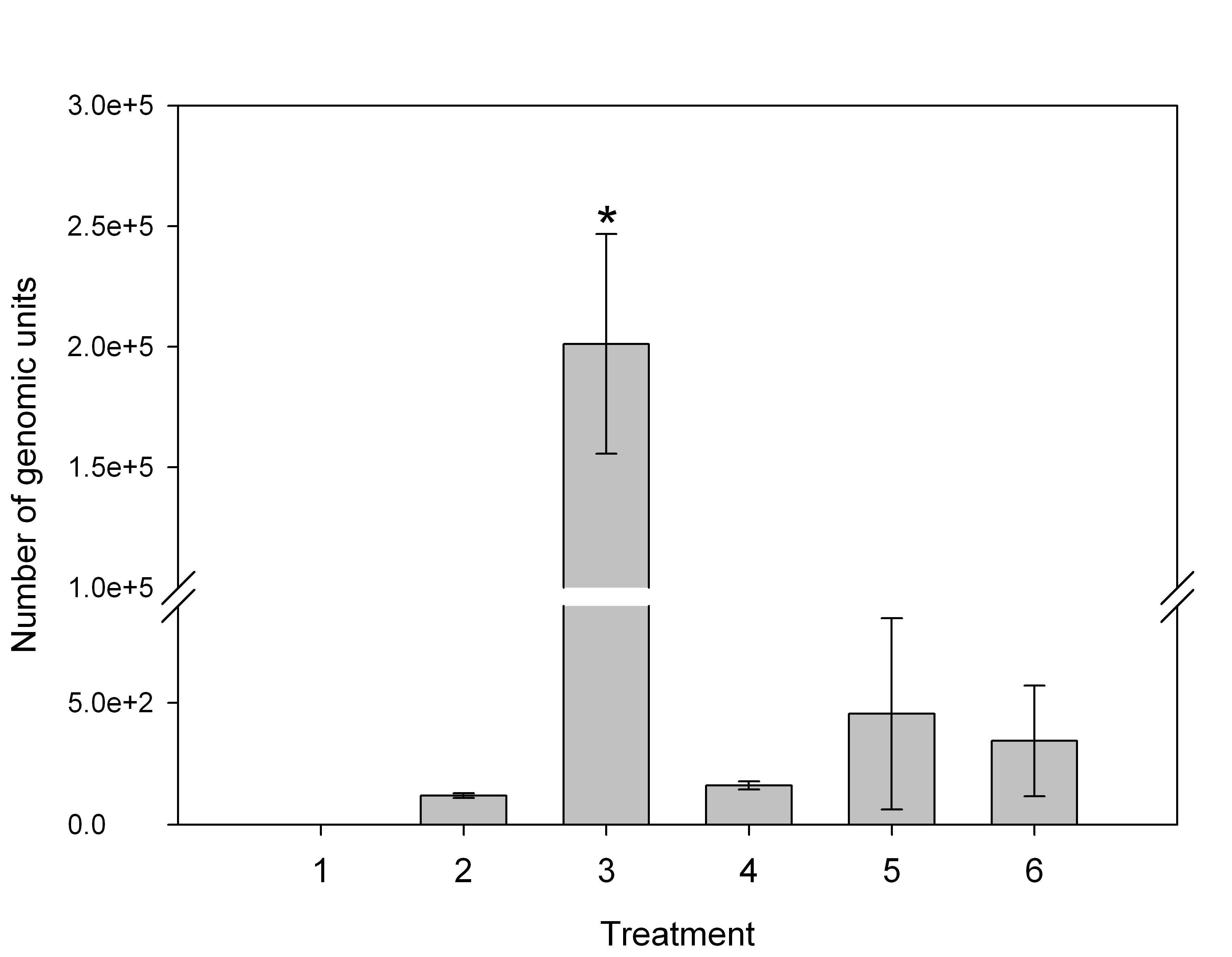

Supplement: Additional file 2: Figure S2 — ToSRV-[BR:PG1:Pep:03] DNA-A accumulation in single or dual infections with ToRMV-[BR:Ub1:96] in tomato plants. Total DNA was extracted from systemically infected leaves at 28 days post-inoculation and used as a template for quantitative, real-time PCR (qPCR) using ToSRV DNA-A primers. 1. Mock-inoculated plants; 2. ToRMV DNA-A + DNA-B; 3. ToSRV DNA-A + DNA-B; 4. ToRMV DNA-A + DNA-B and ToSRV DNA-A + DNA-B; 5. ToRMV DNA-A + DNA-B and ToSRV DNA-A; 6. ToRMV DNA-A and ToSRV DNA-A + DNA-B. In treatment 2, the ToSRV primers weakly amplified ToRMV DNA-A. Values are given as the mean ± confidence interval of three independent tomato plants. Asterisks indicate statistically significant differences by the t test at P ≤ 0.05. [file 1743-422X-11-66-S2.jpeg]
